# Short relative leg length is associated with overweight and obesity in Mexican immigrant women

**DOI:** 10.1186/s12939-019-0988-0

**Published:** 2019-07-03

**Authors:** Mireya Vilar-Compte, James Macinko, Beth C. Weitzman, Carlos M. Avendaño-Villela

**Affiliations:** 1 EQUIDE Research Institute for Equitable Development, Universidad Iberoamericana, Prolongación Paseo de la Reforma 880, Lomas de Santa Fe, 01219 Mexico City, Mexico; 2 Departments of Health Policy and Management and Community Health Sciences, Fielding School of Public Health, UCLA, 650 Charles E. Young Dr. South, 16-035 Center for Health Sciences, Los Angeles, CA 90095-1772 USA; 3 Department of Nutrition and Food Studies, Health and Public Policy, New York University, 411 Lafayette Street, 5th Floor, New York, NY 10003 USA; 4 EQUIDE Research Institute for Equitable Development, Universidad Iberoamericana, Prolongación Paseo de la Reforma 880, Lomas de Santa Fe, 01219 Mexico City, Mexico

**Keywords:** Leg length; obesity, Waist circumference, Body mass index, Stunting, Social mobility in health; social determinants; immigration

## Abstract

**Background:**

Prior research suggests that undernutrition and enteric infections predispose children to stunted growth. Undernutrition and infections have been associated with limited access to healthy diets, lack of sanitation, and access barriers to healthcare – all associated with human rights. Stunting has also been documented to be a major determinant of subsequent obesity and non-communicable diseases. Short leg length relative to stature during adulthood seems to be a good proxy indicator tracking such barriers, and has been reported to be associated with adverse health effects during adulthood. Our objective was to examine the association between relative leg length (as measured by the leg length index, LLI) and measures of adiposity – based on body mass index (BMI) and waist circumference (WC) – in a population of recent Mexican immigrant women to the New York City Area.

**Methods:**

The analysis was based on a cross-sectional survey of 200 Mexican immigrant women aged 18 to 70 years, whose data were collected between April and November 2008; although for purposes of the current study we restricted the sample to those aged 18 to 59 years. The dependent variables were BMI and WC, both transformed into categorical variables. The main independent variable was LLI, and other correlates were controlled for (i.e. age, education, having had children, characteristics of the community of origin, acculturation, chronic conditions, sedentary behaviors, access to fresh fruits and vegetables). Two probit models were estimated: the first one analyzed the effect of LLI on BMI categories and the second one estimated the effect of LLI on WC.

**Results:**

The probit assessing the effect of LLI on overweight/obesity suggested that having a short LLI increased the probability of overweight/obesity by 21 percentage points. Results from the probit model estimating the effect of LLI on WC indicated that having a short LLI increased the probability of having abdominal adiposity by 39 percentage points. Both results were statistically significant at *p* < 0.05.

**Conclusion:**

The study found an association between having shorter legs relative to one’s height and increased risk of overweight/obesity and abdominal adiposity. Findings support the epidemiological evidence regarding the association between short leg length, early life socioeconomic conditions (i.e. limited access to basic rights), and increased risk of adverse health effects later in life.

**Electronic supplementary material:**

The online version of this article (10.1186/s12939-019-0988-0) contains supplementary material, which is available to authorized users.

## Introduction

In order to achieve the United Nation’s (UN’s) Sustainable Development Goals (SDGs), the world requires healthy and productive individuals that have the skills and motivation needed to implement future policies aimed at achieving social and economic development among countries [1]. An adequate nutrition during the first 1000 days of life – from pregnancy until the child’s second year of life – is crucial for children’s development and has important long-term health effects on the well-being of individuals [1]. Undernutrition before birth and during the first 2 years, is known to be a major determinant of stunting in children and plays an important role in the development of subsequent non-communicable diseases and obesity [2]. Prior literature has also documented the existence of “vicious cycles of diseases of poverty” among socially disadvantaged individuals in developing countries [3]. In particular, Guerrant et al. argue that, mainly as a consequence of lack of sanitation and lack of healthcare access, poor children are frequently afflicted by long-lasting enteric infections during their first years 2–3 years of life. The occurrence of these infections, along with the economic barriers that their families face to access healthy foods, predispose poor children to malnutrition and stunted growth. In turn, stunting by 2 years of age, which has been linked to impaired cognitive development, results in the generation of reduced human capital and lower productivity levels in adulthood. Moreover, there is evidence that stunting and enteric infections in malnourished children are associated with the development of obesity, hypertension, diabetes, and other non-communicable diseases in later stages of life, which, in turn, are associated with growing healthcare expenditures that contribute to poverty in vicious cycles [3].

The international human rights law is grounded on The Universal Declaration of Human Rights (proclaimed in 1948), which recognizes that “basic human rights are inherent to all human beings, inalienable and equally applicable to everyone”, regardless our nationality, language, gender, ethnicity, religion, or place of residence. This declaration has been translated into law taking the form of treaties, regional and domestic agreements, and human rights bills through which human rights are expressed and guaranteed [4]. The list of human rights is extensive and includes, among others, the right to health, the right to adequate food, the right to an adequate standard of living, and the right to safe drinking water and sanitation. Because human rights are crucial for the realization of sustainable development, the SDGs that have been adopted by the UN’s 2030 Agenda for Sustainable Development are based on the international human rights law. Therefore, the 17 SDGs are closely related to human rights [5]. For example, SDG 1 (i.e. end poverty in all its forms everywhere) is related to the right of an adequate standard of living; SDG 2, which aims to end hunger, achieve food security and improved nutrition is associated with the right to adequate food; SDG 3 (i.e. ensure healthy lives and promote well-being for all at all ages) relates to the right to health; and SDG 6, which targets the availability and sustainable management of water and sanitation for all, is closely tied to the right to health and to the right to safe drinking water and sanitation.

The vicious poverty cycles outlined above highlight potential barriers to some human rights: the right to an adequate standard of living, the right to equality, the right to safe drinking and sanitation, the right to adequate food, and the right to health. These barriers could potentially jeopardize the realization of the right to health during adulthood and could limit an important mechanism of social mobility (i.e. good health).

From an epidemiological perspective, one of the challenges is to find biological markers in adults that may allow tracing such adverse early environments in childhood. Relative leg length in adulthood^1^ has been proposed as a good marker of childhood conditions, as it is an indicator of pre-pubertal growth. [6–8] During childhood, legs grow rapidly in length and contribute more to variability in stature than does trunk size. [8] Hence, leg length relative to adult stature may detect influences of the nutritional and sanitation environments, as well as the existence/absence of healthcare access barriers during childhood (i.e. relatively longer legs would be associated with a positive nutritional environment, better sanitation, and good access to healthcare, while relatively shorter legs may indicate negative nutritional environments, unfavorable sanitation conditions, and limited access to healthcare services.)

Past studies indicate that adult differences in relative leg length are associated with the risk of cardiovascular disease, insulin resistance, adiposity, and type 2 diabetes, T2D [7–11]. This has been associated, amongst others factors, with different metabolic pathways and epigenetic responses linked to the reduction of fat oxidation which, in turn, could be related to negative environments experienced during early childhood [12]. In particular, negative early environments such as poor diets have been found to be associated with body fatness through three pathways that stimulate the body to store excess fat: low fat oxidation, [8] impairments in appetite control, [13] and physiological mechanisms oriented at improving energetic efficiency [8]. In addition, lack of sanitation and poor access to healthcare services during infancy have also been associated with stunting, thus following similar pathways to stimulate body fatness in adulthood.

From the above evidence, relative leg length seems to be a valid indicator to trace early inequalities, and may help strengthen the argument about the need to invest in policies targeting food security and access to health services during early childhood.

Considering the evidence on the relationship between relative leg length and childhood nutritional environments, the purpose of this study is to determine whether differences in proxy measures of adult adiposity (i.e. body mass index and waist circumference) are associated with relative leg length among a sample of Mexican-born adult immigrant women in the United States (U.S.) The advantage of selecting this sample lies on the fact that in middle-income countries like Mexico, as well as in groups of immigrants of low socioeconomic status in high-income countries, overweight and obesity have been reported to co-exist with short stature and undernutrition [12, 14] . In addition, Mexicans, as well as Mexican immigrants in the U.S. have experienced a striking increase in the prevalence of overweight and obesity during the last decades. Recent national data show that in 2016, 75.6% of women and 69.4% of men over 20 years were reported to be either overweight or obese, making Mexico the country with the second highest prevalence of overweight and obesity in the world [15] . In the U.S., between 1988 and 94 and 2015–16 the prevalence of obesity among Mexican American women increased from 35.4 to 52.3% and among men from 23.9 to 46.2% [16]. Paired with such trends, is the fact that child undernutrition is also prevalent among these populations. In Mexico in 2012, 23.3% of children under 5 years of age suffered from anemia and approximately 21% of the children in rural areas were diagnosed with stunting [17, 18]. In addition, studies have shown that Mexican-born immigrants to the United States are less likely to access and utilize health services, compared to other racial groups in the United States [19]. Similarly, recent evidence from the U.S. suggests that both the prevalence of stunting and malnutrition are higher among hispanic children compared to that of other ethnic groups [20]. Hence, in light of prior evidence regarding stunting and subsequent obesity [2, 3, 7, 8], and because it is likely that some of these immigrants may have been members of socially disadvantaged families and subject to adverse childhood experiences including deficient nutritional environments, lack of sanitation and lack of access to healthcare services [12], this sample of Mexican immigrant women seem to be a relevant population in which the proposed hypothesis (i.e. whether shorter relative leg length is associated with adult adiposity) should be studied, as the cycles of poverty could be replicating and affecting the realization of the right to good health and social mobility in these mothers’ offspring [12]. Therefore, this study aims to examine the association between relative leg length and overweight/obesity in a population of Mexican immigrant women to the New York City area. To our knowledge, this is the first study that explores the role of early childhood stunting on the development of subsequent overweight/obesity among Mexican immigrants who recently migrated to the United States.

## Methods

### Selection of participants

**Fig. 1:**
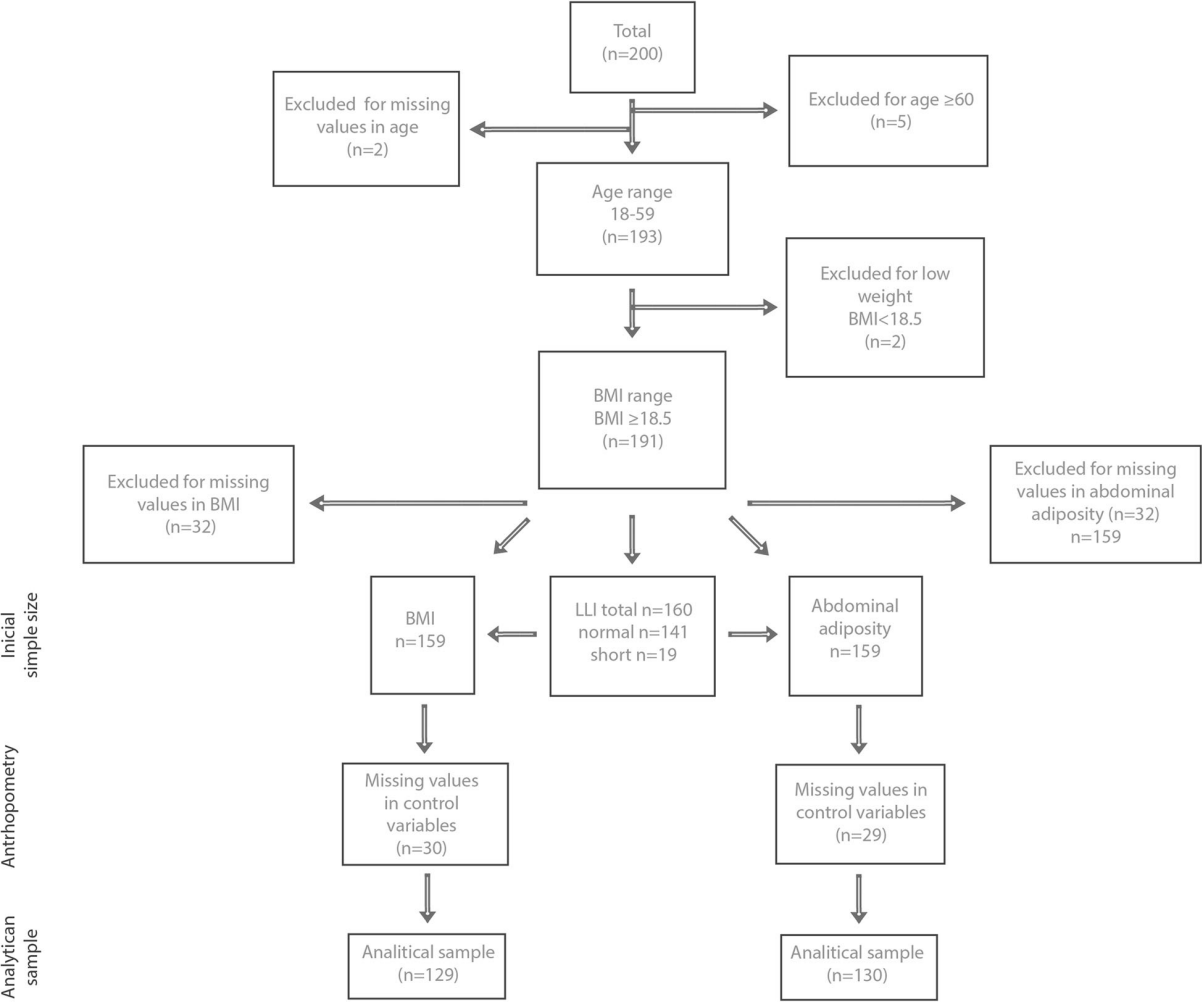
Derivation of Analytical Samples

This study used cross sectional data of women from Puebla State, Mexico who had recently migrated to the New York City Area (NYCA) mostly from 20 specific communites,^2^ that were classified as having relatively high poverty levels. Data were collected from women doing their paperwork at the General Consulate of Mexico in New York City between April and November 2008 for a study that aimed to examine the effects of migration to the NYCA on relative weight and health-behaviors in a group of recent Mexican immigrant women. Eligibility criteria in the original study included female immigrants from Puebla, 18 years or older and not pregnant at the time of interview. Subjects’ age ranged from 18 to 70 years. However, for purposes of the current analysis, we only kept those in the age range 18–59 years. On average, the women in the study had migrated from Puebla 11 years earlier. In the original study, a total of 371 women agreed to participate. The original design of the study contemplated a larger n. In particular, the sampling size was computed using Epi Info 3.2 (please refer to the Additional file 1: Table S1A for the calculation of n). However, at the end of the data collection period, the number of Mexicans entering the Mexican Consulate diminished enormously. The exact causes are unknown but it coincides with the financial crises in 2008. Since women entering the Consulate at this last stage could have been different in ways affecting the study, it was decided to rely on a smaller n despite its potential impact on data analysis. From these initial 371 women, 171 of them did not fulfill the eligibility criteria mostly because they were not from Puebla State and in fewer occasions because they were pregnant or breastfeeding. From the remaining 200 women and for purposes of the present study, a total of 9 women were dropped: 5 of them because they were 60 or older, 2 of them had low values for BMI and an additional 2 women were dropped because they had missing values for age. Therefore, the initial sample size in this study consisted of 191 immigrant women. The derivation of the analytical sample sizes is presented in Fig. 1.

The Consulate was chosen as a recruitment site since it is expected that almost all immigrants – documented and undocumented – would visit the Consulate during their stay in the U.S. In 2008, the Consulate reported it had issued 189,000 passports and *matriculas consulares*.While the passports are a common ID for documented immigrants, *matriculas* are IDs issued by the Mexican Government through its diplomatic missions in the U.S. to individuals regardless of their migratory status. Although such cards cannot be used as a proof of permission to reside or work in the U.S., they are accepted by many local banks, law enforcement agencies and local governments to establish holders’ local address, making them essential, especially for undocumented immigrants [21]. Therefore, the use of this venue sought to reduce bias that would be introduced by recruiting participants from health or community centers.

Participation rate among women in the initial study approached 81%. Missing responses were mostly a result of women finishing up their paperwork at the Consulate before the questionnaire was completed. New York University’s Institutional Review Board approved the protocol for primary data collection.

### Measures

#### Anthropometric variables

Height and weight wearing light clothing were measured during the interview by a group of trained interviewers. Height was measured to the nearest 1 mm using a Seca portable stadiometer. A Seca medical electronic scale was used for body weight measurements, which were rounded to 0.1 kg. Body mass index (BMI; kg/m2) was calculated. According to internationally accepted WHO cut-off points, normal weight was defined as a BMI of 18.5 to 24.9, overweight between 25 and 29.9, and obesity as a BMI ≥30.

Waist circumference (WC) was measured to the nearest 1 mm using a Seca non-stretch tape. Participants were asked to stand with feet apart, finding the midway between the uppermost border of the iliac crest and the lower border of the rib cage, placing the tape around the abdomen at such midpoint. Internationally accepted cut-off points for WC have defined ≥88 cm as a proxy indicator of fat accumulation [22, 23]. However, these values may not be uniformly applicable to other ethnic groups due to differences in body composition [24]. Prior studies suggest that lower cut-off points are needed for Mexicans, proposed alternatives range between 80 and 85 cm [24–29]. Evidence drawn from prior research studying WC and its association with metabolic conditions report a WC ≥ 85 cm as abdominal accumulation sufficient to predict metabolic problems (i.e. T2D, hypertension, etc.), hence, the cut-off point for abdominal adiposity was set at 85 cm or more [26].

Leg length measures are composed of two anatomical parts – tibia and femur. Most studies have estimated relative leg length based on both parts. However, when assessing populations with high prevalence of overweight and obesity, measuring the femur may bias the estimated size of the leg, as the gluteus-femoral fat mass may impair adequate assessment [13]. Therefore, we use a surrogate measure of lower leg length (i.e. tibia, knee height). In addition, measures of lower leg length have been reported to be more sensitive to early environmental influences than those including the femur, so when attempting to capture factors related to the environment rather than heritability, measuring the lower leg may be preferable [30].

Lower leg length was recorded through standing knee height, measured with the subject standing with their heels together, wearing no shoes and weight equally distributed. The center of the knee cap of the right leg was located, a line was drawn in the lateral portion of the leg, and the distance from this mark to the floor was measured using a non-flexible Seca tape. While the common measure of lower leg length is based on knee height taken on a supine or sitting position, the adaptation of standing knee height was decided upon field conditions and following suggested ergonomic standards ( Additional file 1: Figure S1 ). Field conditions implied restrictions in terms of space and time, as subjects were surveyed while on line to complete their paperwork at the Consulate. Technicians were trained and standardized to take alternative knee-height measures while participants were queuing.

Based on Frisancho, the lower leg length index (LLI) was derived from the lower leg length by computing equation (1) [8]. It is important to highlight that the index is statistically valid as it estimates the ratio of a portion of the leg (equally measured in the study population) divided by the height.

Lower leg length index (%) = [(lower leg length cm /stature cm)*100] = relative leg length*100………..(1)

As suggested by Frisancho [8], values of LLI were standardized by converting them to z-scores. Subsequently, a dichotomous variable was created. Short LLI was defined as z-score below a cut-off point of 10%. The decision to use this cut-off point was based on the characteristics of the sample (e.g. documentation status and poverty indexes in the womens’communities of origin). Based on this cut-off point, the dichotomous variable took the following values: 1 = short leg length, 0 = non-short leg length.

Building from the available literature, a conceptual model explaining weight and adiposity was constructed. This model led to including the variables mentioned below, all of which are commonly included in studies analyzing determinants of overweight/obesity and abdominal adiposity.

#### Contextual factors

Characteristics of the sending community were obtained by asking participants to provide the name of the community where they lived before migrating. This community was matched to indicators from the *Consejo Nacional de Población* (CONAPO), which provides total population for each Mexican community based on Census data. Communities with a population below 5000 were defined as rural, communities with a population between 5000 and 14,999 were defined as semi-urban, and communities with a population of 15,000 or greater were classified as urban [31].

To measure subjects’ perceived availability and accessibility of healthy foods in their community, interviewers inquired about the perceived relative price of fresh fruits and vegetables compared to other food choices in the community at the time of the interview (this variable was dichotomized as 1 = affordable, 0 = more expensive) [32, 33].

#### Acculturation

The brief Acculturation Rating Scale for Mexican Americans-II (ARSMA) was used. This validated scale has 12 items, 6 for the Anglo orientation scale (AOS) and 6 for the Mexican orientation Scale (MOS), both of which are independently derived [34, 35]. Items are based on a Likert-type scale that measures acculturation along three primary dimensions: language, ethnic identification, and ethnic integration. Acculturation level was computed as [SUM(AOS)/6]-[SUM(MOS)/6]. An orthogonal acculturation index was calculated to describe acculturative types. Since a small proportion of the sample was categorized as high bicultural or assimilated, the acculturation measure was transformed into a dichotomous variable categorizing participants as being traditional (i.e. if MOS ≥ 3.7 and AOS ≤ 3.24) or not (i.e. if MOS < 3.24).

#### Health and lifestyle variables

Self-reported diagnosis of depression, high blood pressure, cancer, asthma/bronchitis, cardiovascular diseases or T2D were recorded. Self-reporting any of these conditions, led to identifying the participant through a dichotomous variable when having at least 1 chronic condition.

As a proxy for sedentary behaviors, participants were asked to report number of hours spent in 1 week watching TV. Those reporting 8 or more hours were classified as sedentary.

#### Other covariates

Age, age squared, education and having had children were included as socio-demographic indicators. Age was measured as a continuous variable; the model also included age squared – a common statistical transformation to account for relationships that are not linear, hence, implying that the relationship between age and measures of adiposity varies across the lifespan. Education was categorized as 0 to 6 years, 7 to 12 years, and 12 years or more of formal education. Having had children was transformed as a dichotomous variable.

### Statistical analysis

Statistical analyses were conducted using Stata (v. 13.1) [36]. Descriptive analyses were conducted for anthropometric and socio-demographic variables. Then two regression models were estimated to assess the association of LLI and proxy measures of adiposity. A probit regression model estimated the association between LLI and BMI categories, while a binomial probit regression assessed the relationship between LLI and abdominal adiposity. Marginal effects were computed for both regressions. The Stata command *mfx* was used to obtain the marginal effects, which are the partial derivatives of the predicted probability with respect to the independent variables. Hence, they are estimated to assess the contribution of each independent variable to the probability of being normal weight, overweight or obese, in the case of categorical BMI. Similarly, they assess the contribution of each independent variable to having or not abdominal adiposity when WC measure is used as a dependent variable. Extending these analyses, predictive margins of LLI interacted with characteristics of the community of origin were computed both for categorical BMI and abdominal adiposity.

Those results with *p* < 0.05 are defined as statictically significant (in addition, *p*-values are specified in each Table).

The datasets used and/or analysed during the current study are available from the corresponding author on reasonable request.

## Results

As shown in Table 1, participants were on average 32.9 years old. Most participants had between 6 and 12 years of education (69.6%), and 88.0% reported having had at least one child. One third of the sample (31.9%) reported having at least one chronic condition. More than half of the sample migrated to the NYCA from urban communities in Mexico (54.2%), about one-third (31.6%) migrated from semi-urban areas, and less than one sixth (14.3%) did so from rural communities. Most women were not acculturated; 74% of them were categorized as traditional. About half of the sample (49.1%) was categorized as sedentary, and more than two-thirds (67.4%) of respondents thought that fresh fruits and vegetables were more expensive than other food choices in their communities (Table 1).

**Table 1:** Descriptive Statistics of Survey Participants

	Total 191	Short 19	Normal 149	P-value
Anthropometry				
Standing knee height, mean (SD)	42.5 (3.2)	37.6 (1.3)	43.1(2.8)	0.00^a^
Body Mass Index (BMI), mean (SD)	28.1 (4.7)	29.2 (4.3)	28.0 (4.8)	0.31^a^
BMI categories %, n				
Normal weight (18.5 ≤ 24.9 kg/m^2^)	27.0 (43)	15.8 (3)	28.6 (40)	0.43^c^
Overweight (25 ≤ 29. 9 kg/m^2^	41.5 (66)	52.6 (10)	40.0 (56)	
Obese (≥ 30 kg/m^2^)	31.5 (50)	31.6 (6)	31.4 (44)	
BMI categories 2%, n				
Normal weight (18.5 ≤ 24.9 kg/m2)	27.0 (43)	15.8 (3)	28.6 (40)	0.28^c^
Overweight and Obese (≥ 25 kg/m2)	73.0 (116)	84.2 (16)	71.4 (100)	
WC, mean (SD)	88.9 (12.2)	93.9 (11.0)	88.3 (12.3)	0.06^a^
WC categories %,n				
Not at-risk	37.1 (59)	15.8 (3)	40.0 (56)	0.04^c^
Higher-risk	62.9 (100)	84.2 (16)	60.0 (84)	
Height, mean (SD)	151.7 (5.4)	151.7 (4.0)	151.8 (5.6)	0.95^a^
Weight, mean (SD)	64.7 (11.3)	67.0 (9.1)	64.4 (11.5)	0.35^a^
Demographic and contextual factors				
Age, mean (SD)	32.9 (8.5)	33.7 (11.6)	32.8 (8.0)	0.75^a^
Education				
< 6 years	11.5 (22)	5.3 (1)	10.6 (15)	0.79^c^
6 to 12 years	69.6 (133)	79.0 (15)	68.8 (97)	
> 12 years	18.9 (36)	15.8 (3)	20.6 (29)	
Having had children %,n (Yes)	88.0 (168)	84.2 (16)	87.2 (123)	0.71^c^
Having at least 1 chronic condition (Yes)	31.9 (61)	36.8 (7)	27.7 (39)	0.40^b^
Community of origin %, n				
Urban	54.2 (91)	58.3 (7)	50.8 (65)	092^c^
Semi-urban	31.6 (53)	33.3 (4)	33.6 (43)	
Rural	14.3 (24)	8.3 (1)	15.6 (20)	
Acculturation (Traditional)	74.0 (128)	72.2 (13)	72.3 (99)	0.99^b^
Perceived relative prices of fresh fruits & vegetables (More expensive)	67.4 (116)	63.2 (12)	67.2 (90)	0.73^b^
Sedentary based on TV watching (Yes)	49.1 (86)	52.6 (10)	50.0 (70)	0.83^b^

Notes: ^a^ A t-test on the equality of means was conducted. ^b^ A Pearson chi-square test was conducted. ^c^ A Fisher’s exact test was conducted

Results from the probit model analyzing the relationship between LLI and BMI categories suggest that short LLI is strongly and significantly associated with a greater probability of being overweight/obese.^3^ In particular, estimated marginal effects show that having a short LLI increased the probability of being overweight/obese by 21 percentage points (p.p). The “protective” effect of migrating from semi-urban and rural areas should be highlighted, as it decreased the probability of overweight/obesity by 29 and 58 p.p, respectively. Being classified as traditional increased the probability of having overweight/obesity by 23 p.p.. In addition, perceiving high prices of fresh fruits and vegetables increased the probability of being overweight/obese by 21.p.p. Having had a children increased the probability of being overweight/obese by 38 p.p. (Table 2).

**Table 2:** Probit model estimating BMI categories (collapsed overweight and obesity)

	Overweight and Obesity			
Variable	Coefficient	95% CI	Marginal effect	95% CI
Age	0.10	(−0.20,0.38)	0.03	(−0.06,0.12)
Age squared	−0.00	(− 0.00,0.00)	− 0.00	(− 0.00,0.00)
Short LLI	0.98†	(− 0.07,2.02)	0.21†	(0.06,0.34)
Education				
< 6 years	–	–	–	–
6 to12 years	0.09	(−0.87,1.05)	0.03	(−0.27,0.32)
> 12 years	−0.08	(−1.16,0.99)	−0.02	(− 0.36,0.31)
Having had children	1.06†	(0.22,1.89)	0.38‡	(0.22,1.89)
Chronic condition	0.03	(−0.69,0.63)	0.01	(−0.21,0.19)
Community of origin				
Urban	–	–	–	–
Semi-urban	−0.91†	(−1.53,-0.28)	−0.29†	(− 0.49,-0.09)
Rural	−1.63*	(−2.55,-0.71)	−0.58*	(− 0.86,-0.30)
Traditional	0.69†	(0.09,1.30)	0.23†	(0.02,0.44)
Fresh fruits & vegetables perceived as more expensive	0.68†	(0.09,1.26)	0.21†	(0.02,0.40)
Sedentary	0.03	(−0.55,0.62)	0.00	(−0.17,0.19)
N	129			

Notes: *CI* confidence interval. Significance *p < 0.001; †*p* < 0.01; ‡*p* < 0.05

The probit model estimating the probability of abdominal adiposity (WC ≥85 cm) led to similar findings (Table 3).

**Table 3:** Probit model estimating abdominal adiposity (> = 85 cm)

Variable	Coefficient	95% CI	Marginal effect	95% CI
Age	0.03	(−0.25,0.30)	0.01	(−0.08,0.10)
Age squared	0.00	(0.00,0.00)	0.00	(−0.00,0.00)
Short LLI	1.19†	(0.14,2.24)	0.39*	(0.14,0.47)
Education				
< 6 years	–	–	–	–
6 to12 years	−0.01	(− 0.91,0.89)	−0.00	(− 0.32,0.32)
> 12 years	− 0.27	(−1.28,0.74)	−0.10	(− 0.48,0.29)
Having had children	0.65	(−0.14,1.44)	0.25	(−0.05,0.55)
Chronic condition	1.04†	(0.34,1.74)	0.32*	(0.15,0.48)
Community of origin				
Urban	–	–	–	–
Semi-urban	−0.83†	(−1.40,-0.27)	− 0.30†	(− 0.51,-0.10)
Rural	− 0.98†	(− 1.81,-0.15)	−0.37†	(− 0.67,-0.07)
Traditional	0.25	(−0.33,0.83)	0.09	(−0.12,0.30)
Fresh fruits & vegetables perceived as more expensive	0.68†	(0.14,1.22)	0.25†	(0.05,0.45)
Sedentary	0.00	(−0.57,0.56)	0.00	(−0.20,0.20)
N	130			

Notes: *CI* confidence interval. Significance *p < 0.001; †*p* < 0.01; ‡*p* < 0.05

Marginal effects suggest that having a short LLI increased the probability of having a abdominal adiposity WC by 39 p.p.. The model also highlighted the seemingly “protective” effect of migrating from semi-urban and rural communities, as they were associated with a lower probability of having an abdominal adiposity by 30 and 37 p.p., respectively. Perceived higher prices of fruits and vegetables were associated with a 25 p.p. probability increase of abdominal adiposity (Table 3). In addition, Table 3 shows that, as expected, having a chronic condition was positively associated with the risk of abdominal adiposity by 32 p.p.

**Fig. 2:**
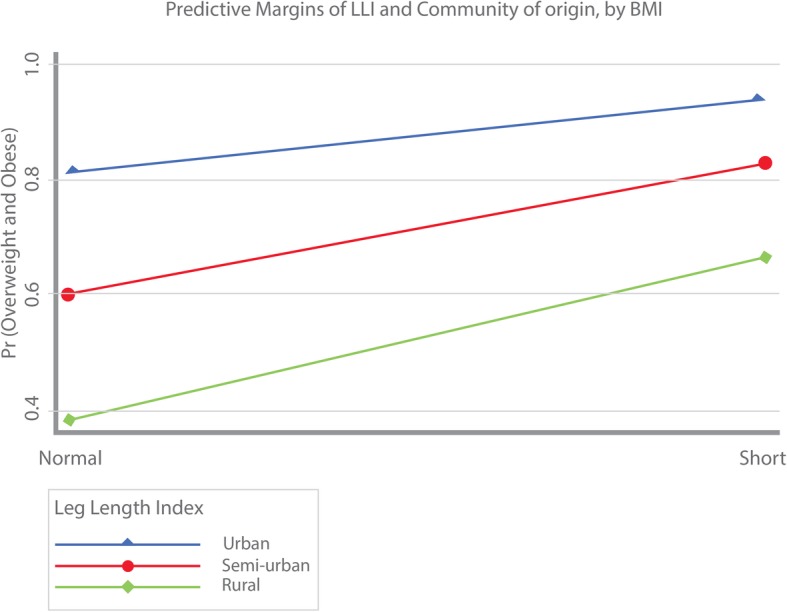
Predictive margins of LLI and community of origin by BMI

**Fig. 3:**
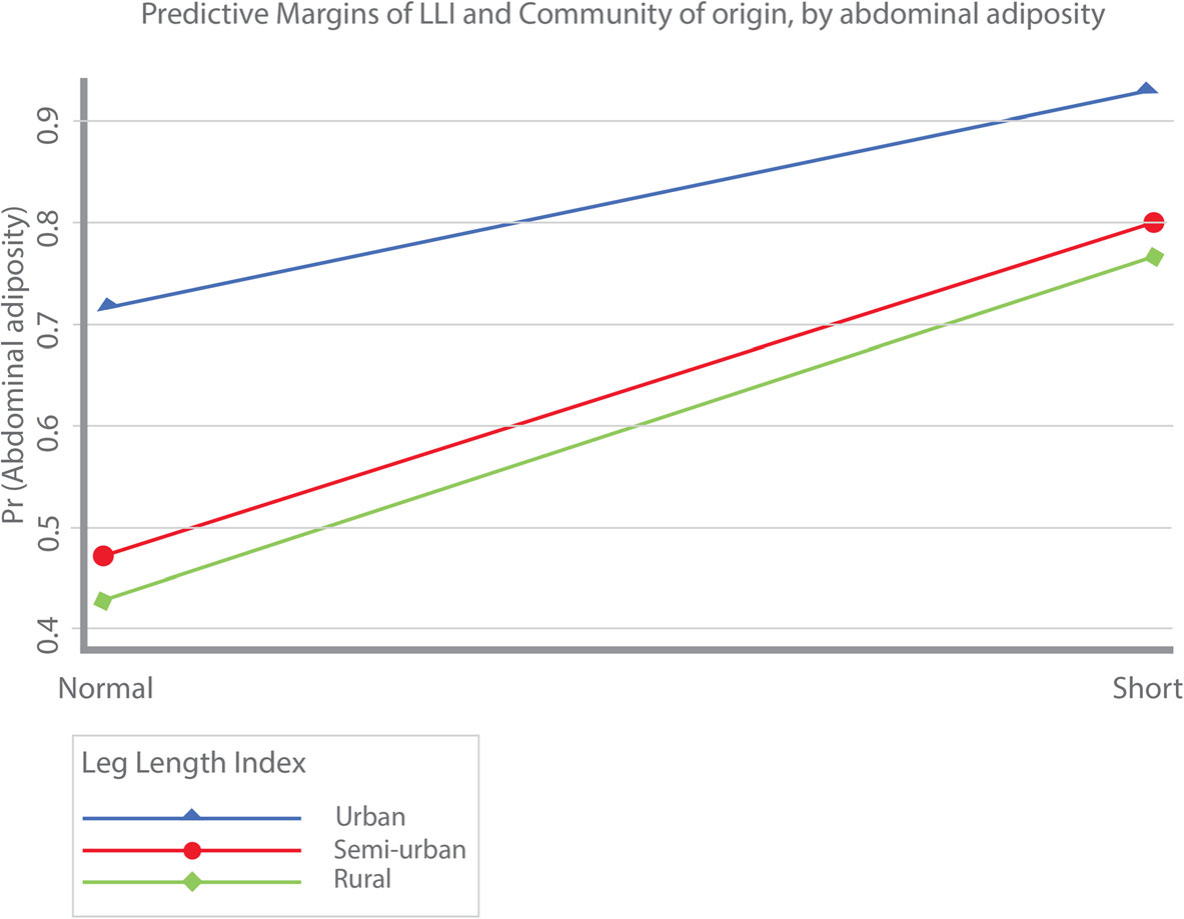
Predictive margins of LLI and community of origin by abdominal adiposity

Due to the associations found between proxy measures of adiposity and rurality, predictive margins of LLI interacted with type of community (i.e. urban, semi-urban and rural) were computed for the probability of overweight/obesity (Fig. 2) and the probability of abdominal adiposity (Fig. 3). As shown, both the probability of overweight/obesity and abdominal adiposity were larger among those with short LLI compared to individuals with normal LLI. In addition, the largest probability of overweight/obesity and abdominal adiposity was greater among urban dwellers, followed by immigrant women from semi-urban areas. Lastly, women migrating from rural areas were associated with the lowest probabilities of overweight/obesity and abdominal adiposity.

## Discussion

Our findings suggest that short relative leg length was strongly associated with 2 different proxy measures of adiposity among a sample of Mexican immigrant women who had recently migrated to the NYCA. Significant associations between short LLI and proxies of adiposity were found even after controlling for several important covariates that have been associated with an increased risk of obesity. The association of short LLI with proxy measures of adiposity was consistent in both models (i.e. BMI and WC). Therefore, our results support the hypothesis that early childhood stunting is associated with adult adiposity. Because these women migrated mainly from disadvantaged communities in Puebla, it is highly likely that stunting could have been the result of adverse economic childhood environments associated with lack of sanitation, lack of healthcare access, infectious diseases, and malnutrition, which could, in turn, limit the realization of certain human rights (i.e. right to an adequate standard of living, the right to equality, the right to safe drinking and sanitation, the right to adequate food, and the right to health). Altough we could not control for how long the women lived in the country of origin, they did not report in the survey any prior migration process (this was asked openly). Furthermore, because stunting during early stages of life has been associated with the development of hypertension and diabetes, it is likely that the immigrant women with short LLI who now live in the U.S. (most of whom are undocumented), may be facing health care access barriers and increasing healthcare expenditures, which contribute to impoverishment, thus closing the “vicious cycles of diseases of poverty” and perpetuating the barriers to human rights.

Findings regarding rurality were unexpected because rurality is commonly associated with higher prevalence of undernutrition, poverty, and lower access to healthcare services [37]. However, in our analysis, rurality contexts of origin appear as a factor that reduces the probability of overweight/obesity and abdominal adiposity. Although short relative leg length in our sample was associated with a greater risk of adiposity in all women (regardless of the type of community of origin), we observed that women with short relative leg length that migrated from urban or semi-urban communities had a higher risk of adiposity, compared to their rural counterparts. This was confirmed by an interaction analysis between LLI and rurality. In addition, results from such analysis revealed that migrant women from urban and semi-urban communites, regardless of their LLI status, were associated with higher probabilities of abdominal adiposity and higher risks of overweight/obesity, compared to migrant women from rural communities. These finding could be in line with research that suggests that the rapid urbanization process taking place in middle income countries such as Mexico has fueled rising urban poverty rates compared to those in rural areas [38]. These increasing poverty rates could, in turn, be related to malnutrition (e.g eating ultra-processed foods) and poor health among urban settlers. In addition, this result could be associated with the literature that sustains that the apparent “urban vs. rural advantage” is often masked by urban averages that conceal significant health inequities within urban populations [39]. In sum, these findings leave interesting questions for future research, especially regarding the effect of the built environment on adiposity, as well as potential biases brought by different migratory processes. Future studies should profit from longitudinal data to address trajectories that analyze the association of early life environments with LLI and the subsequent increased risk of adiposity during adulthood.

There are some limitations linked to the cut-off points selected for some of our anthropometric measures. First, in prior research, short LLI has been defined as the lower 5% of the z-score distribution [8, 30]. However, our cut-off point was set at 10%. As mentioned before, this decision was based on the characteristics of the sample. Most of the women interviewed were undocumented immigrants to the U.S, and more than 70% migrated from communities of origin with medium, high or very high poverty indexes, hence, potentially skewing the distribution of LLI. To assess this bias we ran sensitivity tests (results not shown) that found significant effects up to the cut-off point of 15%. Second, there is a debate in terms of how leg length should be measured. We decided to use lower leg length measures to avoid measurement biases that fat accumulation can bring in the thigh section [30]. In addition, our measure of lower leg length is based on a surrogate measure of knee height – i.e. standing knee height – that was decided upon field conditions previously described. This measure is not the gold standard in anthropometry, but the potential measurement errors are assumed to be distributed in the sample as the technicians were standardized and all subjects were measured through the same procedure. In addition, z-scores were calculated using the sample population, thus, implying that the LLI computed is robust within the sample as it accounts for a measure of the same leg segment of the body for the population studied. However, in future studies conventional knee height measure (i.e. in supine or sitting position) should be tested. Additional limitations of the study include the use of self-reported health conditions and the small sample sizes. The bias introduced by self-reported health conditiotions could have resulted in lower prevalences of such conditions. However, this is common in population studies, and has been widely reported. Furthermore, the small sample sizes employed in the study could also raise concerns. However, we believe that finding statistically significant effects of LLI on two different proxies for adiposity by using such small samples is surprising. Finally, since the sample refers to a very specific population (i.e. Mexican immigrant women who recently migrated to the NYCA), this may compromise the external validity of this study.

## Conclusions

Short LLI is associated with proxy measures of adiposity among a sample of Mexican immigrant women, even after controlling for important confounders. However, results of the study should be interpreted with caution as they refer to a very specific population (i.e. Mexican immigrant women to the NYCA). Findings were consistent with two different proxies of adiposity (i.e. BMI and WC), and support prior evidence suggesting that relative short leg length is a valid indicator of increased risk for obesity and other chronic conditions in adulthood. In addition, short relative leg length in adulthood may be an important proxy indicator of unequal access to food, sanitation and health. As mentioned, inequalities in early life are associated with consequences in adulthood and may threaten the right to health and the probability of social mobility across generations. Furthermore, it is well known that conditions of poverty and inequality underlie the search for new opportunities through migration from the South to the North. However, these conditions may lead to accumulated health risk processes later in life as a consequence of the lack of effective access to basic rights during childhood. Such lack of access and the subsequent metabolic effects in life, highlight the relevance of investing in polices targeting the first 1000 days of life, securing food security and access to health care. These are key interventions highlighted in the SDGs and will be enormously important to improve the effective right to health in low- and middle- income countries, such as Mexico.

## Additional file

Additional file 1: **Table S1.**A Calculation of simple size in original 2008 study. **Table S1.**B Multinomial probit model estimating BMI categories. **Figure S1.** Standing knee-height measurement according to standards from the University of Michigan (HUMOSIM Anthropometric Measurements, 2003). (DOCX 53 kb)

## Data Availability

The datasets generated during the current study are available from the corresponding author on reasonable request.

## Abbreviations

AOS: Anglo Orientation Scale
ARSMA: Acculturation Rating Scale for Mexican Americans
BMI: Body Mass Index
CONAPO: Consejo Nacional de Población
LLI: Leg Length Index
MOS: Mexican Orientation Scale
NYCA: New York City Area
SDG: Sustainable Development Goal
T2D: Type 2 Diabetes
U.S.: United States
UN: United Nations
WC: Waist Circumference
WHO: World Health Organization

## Footnotes

1 Refers to leg length, relative to an adult’s stature. Relative leg length is usually operationalized by the leg length index (LLI).

2 Approximately 60–80% of all Mexican New Yorkers at the time of the study had recently migrated (i.e. on average, 11 years earlier) from around 20 common localites in Puebla, Mexico, in the early years of the twenty-first century. Such clustered migratory processes (both at the state of Puebla and within Puebla) provides a very specific phenomenon that reduces bias that might be introduced by unmeasured genetic, geographic or cultural influences often present in studies that group immigrants from different regions [40, 41].

3 An additional multinomial probit model in which overweight and obesity categories was implemented. Results were similar to those of the probit model in which overweight and obesity categories were collapsed (please refer to the Additional file 1: Table S1B section.)
