# Supplementary material for: Short relative leg length is associated with overweight and obesity in Mexican immigrant women
Source: Int J Equity Health. 2019 Jul 3;18:103. doi: 10.1186/s12939-019-0988-0 (PMC6609393; doi:10.1186/s12939-019-0988-0)
Supplement: Supplementary file 1 — Table S1.A Calculation of simple size in original 2008 study. Table S1.B Multinomial probit model estimating BMI categories. Figure S1. Standing knee-height measurement according to standards from the University of Michigan (HUMOSIM Anthropometric Measurements, 2003). (DOCX 53 kb) [file 12939_2019_988_MOESM1_ESM.docx]

**Additional file 1**

**Figure S1. Standing knee-height measurement according to standards from the University of Michigan (HUMOSIM Anthropometric Measurements, 2003)**

Have the subject stand erect with their heels together and weight distributed evenly between both feet. Locate the patella (kneecap) on the front of the knee and find the center of that bone. Measure the distance from the floor to this location using a caliper or a flexible tape.


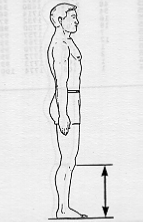


**Table S1A: Calculation of simple size in original 2008 study**

| Sample size |  | 277 |
| --- | --- | --- |
| Adjusted sample size |  | 332 |
| Proportion top 20 communities (w)* | 4,998/39,728 | 0.13 |
| Systematic random sampling interval | 4,998/332 | 15 |
| Weighted interval | (15) (0.13) | 2 |

*Based on information provided by the Mexican General Consulate in NYC

**Table S1B. Multinomial probit model estimating BMI categories**

|  | Overweight | | | Obesity | | |
| --- | --- | --- | --- | --- | --- | --- |
| Variable | Coeff. | 95% CI | Coeff. | | 95% CI |  |
| Age | 0.08 | (-0.32,0.48) | | 0.31 | (-0.18,0.79) | |
| Age squared | -0.00 | (-0.01,0.01) | | -0.00 | (-0.01,0.00) | |
| Short LLI | 1.10 | (-0.36,2.53) | | 1.45‡ | (-0.13,3.03) | |
| Education  <6 years  6 to12 years  >12 years | --  0.41  0.13 | --  (-0.98,1.79)  (-1.42,1.68) | | --  -0.40  -0.64 | --  (-1.81,1.01)  (-2.26,0.98) | |
| Having had children | 1.10‡ | (-0.10,2.26) | | 2.00† | (0.44,3.52) | |
| Chronic condition | -0.53 | (-1.49,0.43) | | 0.58 | (-0.40,1.55) | |
| Community of origin |  |  | |  |  | |
| Urban | -- | -- | | -- | -- | |
| Semi-urban | -1.00† | (-1.91,-0.16) | | -1.50* | (-2.50,-0.52) | |
| Rural | -1.90* | (-3.17,-0.61) | | -2.30* | (-3.68,-0.89) | |
| Traditional | 0.98 † | (0.11,1.84) | | 0.75 | (-0.21,1.72) | |
| Fresh fruits & vegetables perceived as more expensive | 0.59 | (-0.24,1.42) | | 1.55* | (0.56,2.54) | |
| Sedentary | -0.15 | (-0.98,0.68) | | 0.16 | (-0.76,1.09) | |
| N | 129 |  | |  |  | |
| Notes: CI, confidence interval. Significance *p<0.001; †p<0.01; ‡p<0.05. | | | | | | |
